# Association between serum 25-hydroxyvitamin D and global DNA methylation in visceral adipose tissue from colorectal cancer patients

**DOI:** 10.1186/s12885-018-5226-4

**Published:** 2019-01-21

**Authors:** Daniel Castellano-Castillo, Sonsoles Morcillo, Ana B. Crujeiras, Lidia Sánchez-Alcoholado, Mercedes Clemente-Postigo, Esperanza Torres, Francisco José Tinahones, Manuel Macias-Gonzalez

**Affiliations:** 1 Unidad de Gestión Clínica Endocrinología y Nutrición. Instituto de Investigación Biomédica de Málaga (IBIMA), Complejo Hospitalario de Málaga (Virgen de la Victoria) Málaga (Spain). CIBER Fisiopatología de la Obesidad y Nutrición (CB06/03), Málaga, Spain; 2 Epigenomics in Endocrinology and Nutrition Group. Instituto de Investigacion Sanitaria (IDIS), Complejo Hospitalario Universitario de Santiago (CHUS) and CIBER Fisiopatologia de la Obesidad y Nutricion (CIBERobn), Málaga, Spain; 3 Unidad de Gestión Clínica de Oncología Intercentros Hospital Universitario Virgen de la Victoria, Málaga, Spain

**Keywords:** Colorectal cancer, Global DNA methylation, Obesity, Epigenetic, Vitamin D

## Abstract

**Background:**

Visceral adipose tissue (VAT) has been identified as the essential fat depot for pathogenetic theories that associateobesity and colon cancer. LINE-1 hypomethylation has been mostly detected in tumor colon tissue, but less is known about the epigenetic pattern in surrounding tissues. The aim was to analyze for the first time the potential relationship between serum vitamin D, obesity and global methylation (LINE-1) in the visceral adipose tissue (VAT) from patients with and without colorectal cancer.

**Methods:**

A total of 55 patients with colorectal cancer and 35 control subjects participated in the study. LINE-1 DNA methylation in VAT was measured by pyrosequencing. Serum 25(OH)D levels were determined by ELISA.

**Results:**

Cancer patients had lower levels of LINE-1 methylation in VAT compared with the control group. In the subjects with colorectal cancer, LINE-1 DNA methylation levels were associated positively with vitamin D levels (*r* = 0,463; *p* < 0.001) and negatively with BMI (*r* = − 0.334, *p* = 0.01) and HOMA insulin resistance index (*r* = − 0.348, p = 0.01). Serum vitamin D was the main variable explaining the LINE-1% variance in the cancer group (β = 0.460, p < 0.001). In a multivariate analysis, subjects with higher LINE-1 methylation values had lower risk of developing colorectal cancer (OR = 0.53; IC95% =0.28–0.99) compared with the control group.

**Conclusions:**

We showed for the first time an association between LINE-1 DNA methylation in VAT and vitamin D levels in subjects with colorectal cancer, highlighting the importance of VAT from cancer patients, which could be modified epigenetically compared to healthy subjects.

## Background

Obesity is considered one of the most important metabolic diseases of this century and is associated with serious comorbidities [1]. Obesity is one of the risk factors that has been linked to the most relevant cancers, like colorectal cancer (CRC) [2]. CRC is the third most common human cancer worldwide [3]. In Spain, the CRC represents the seventh global cause of death in Spain [4]. Epidemiological data suggest that obesity is associated with a 30–70% increased risk of colon cancer in men, whereas the association is less consistent in women [5]. Despite the numerous epidemiological evidences connecting obesity to higher cancer risk and mortality, the possible mechanisms involved are not entirely known [6]. Existing evidence suggest that visceral abdominal fat (VAT) is more predictive of obesity-associated comorbidity and mortality than subcutaneous adipose tissue (SAT) [7]. VAT has been identified as the essential fat depot for pathogenic theories that associate obesity and colon cancer [8]. This has been related to the unique architecture of VAT that is highly cellular, vascular and innervated and contains cells with inflammatory and immunological functions. Moreover, the close proximity of VAT to the portal vein causes drainage of excess free fatty acids and inflammatory mediators directly to the liver, thus affecting metabolism and creating a condition of low grade chronic inflammation, a favorable niche for tumor development [9].

It is suggested that the obesity state provides a particular adipose tissue micro environment with multiple systemic metabolic alterations that contribute to tumor development and progression [6]. All of this through different mechanisms, such as obesity-associated low-grade inflammation, endocrine alterations, insulin resistance, and hypoxia–angiogenesis processes [10].

On the other hand, it is well known the role of epigenetic modifications on cancer development. DNA methylation is the most widely studied epigenetic phenomenon in humans, and the relationship between DNA methylation and carcinogenesis has been extensively studied in human colon cancer [11]. DNA methylation measured by Long Interspersed Nucleotide Element 1 (LINE-1) sequences has been considered a surrogate marker for global genome methylation [12]. LINE-1 hypomethylation has been associated with poor survival and worse clinical outcome in CRC patients [13]. Currently, it is well recognized a global hypomethylation of genomic repetitive sequences in malignant cells, but less is known about the epigenetic pattern in surrounding tissues. Hypomethylation of LINE-1 has been mostly detected in tumor colon tissue, but some authors have also detected hypomethylation in non-tumoral area from cancer patients [14].

Environmental and lifestyle factors may influence on epigenetic mechanisms, such as DNA methylation [15]. The consumption of nutrients, such as folates and alcohol, were associated with DNA hypomethylation of LINE-1 in patients with CRC [16, 17]. Other authors have found that women with a low consumption of fruit affect to the global methylation [18]. We have also reported in a Mediterranean diet study a positive association of the hypomethylation in LINE-1 with the weight loss [19]. Folic acid, vitamin B, green tea, alcohol and recently Vitamin D, are known for their effect on epigenetic regulation [20]. It has been proposed another biomarker of CRC proposed is the CpG island methylator phenotype (CIMP) by hypermethylation [21], but it is not clear the relationship with obesity [22, 23].

The serum level of 25-hydroxyvitamin D [25(OH)D] and excess body weight are related with risk of CRC [24, 25]. Furthermore, vitamin D is able to change the profile of the DNA methylation and histone modifications, modifying the mechanism of action of the oncogenes [26].

In this context, due to the close relationship between obesity and colorectal cancer, it is possible that the VAT from CRC patients could be characterized by a specific epigenetic pattern favorable to the promotion of carcinogenesis. In this context, serum 25(OH)D levels could have a significant effect on DNA methylation profile.

The aim of the study was to evaluate whether in the VAT from colorectal cancer patients there is already a LINE-1 hypomethylation compared to subjects without cancer, and to analyze whether such potential association depends on obesity status or other known variables also related to CRC, as serum Vitamin D levels.

## Methods

### Subjects

This study was conducted in 2 groups with CRC (cases) and without CRC (control) participated in the study. Cases were recruited including all patients undergoing colon cancer during the years 2012–2013. Controls were recruited from patients who underwent hiatus hernia surgery or cholecystectomy between 2012 and 2013 in an operating room in general surgery. All these subjects were over 54 years of age and BMI < 40; this criterion was included to ensure homogeneity in the age and BMI of both groups. Patients were excluded if they had cardiovascular disease, arthritis, acute inflammatory disease, infectious disease, renal disease, were receiving drugs that could alter the lipid or glucose profile, calcium or vitamin D supplements. All the participants were informed of the nature of the study and gave their written consent. The study was approved by the Ethics and Research Committee of the University *Virgen de la Victoria* Hospital in Málaga, Spain.

#### Laboratory measurements

Epiploic visceral adipose tissue (VAT) and blood samples were processed as we have previously published [27], before surgery and were maintained at − 80 °C until analysis. Serum glucose, cholesterol, and HDL cholesterol (HDL-C) were measured in a Dimension auto analyzer (Dade Behring Inc.) by enzymatic methods (Randox Laboratories Ltd.). Insulin was quantified by radioimmunoassay supplied by Bio Source International, Camarillo, CA, USA. High-sensitive C-reactive protein (CRP) was analyzed by enzyme immunoassay kits (BLK Diagnostics, Spain). The homeostasis model assessment of insulin resistance (HOMA-IR) was calculated with the following equation: HOMA-IR = fasting insulin (ìIU/mL) × fasting glucose (mmol/L)/22.5. Serum 25(OH)D levels was determined by enzyme immunoassay (ELISA)Immundiagnostik’s kit.

#### Pyrosequencing

The DNA methylation status was determined by pyrosequencing using the PyromakTMQ96 ID Pyrosequencing System (Qiagen). For the methylation analysis of the LINE-1 we used the primer sequences, as we have previously published. DNA methylation analysis were performed on bisulfite-treated DNA using highly-quantitative analysis based on PCR-pyrosequencing [27]. Unmethylated and methylated DNA were included as controls in each run (New England biolabs), inter-assay precision (%CV) was < 2.5%; intrra-assay (%CV) was < 1.0% [22, 27, 28].

### Statistical analysis

One-way ANOVA and Chi2 test were made for calculation of the statistical difference between the means of the continuous and qualitative variables respectively. A two-sided *P*-value of < 0.05 for statistical significance in all analysis was used. Normal distribution was tested for LINE-1 methylation variable by Kolmogorov-Smirnov test. LINE-1 methylation variable was normally distributed. Multiple linear regression analysis were performed to evaluate the potential predictors of LINE-1 methylation variation. A multivariate logistic regression model was performed to evaluate whether global methylation levels can predict CRC risk. Odds ratio (OR) and 95% confidence intervals were estimated by logistic regression. With the sample size of this study we are be able to detect as significant a difference of less than one percentage unit with a statistical power greater than 90%. Statistical power was calculated with Openepi program [29]. All of analysis were performed using R statistical software, version 2.8.1 (Department of Statistics, University of Auckland, Auckland, NZ; http://www.r-project.org/).

## Results

### Characteristics of the study subjects

The characteristics of the subjects are shown in Table 1. Subjects were classified as the control group and the colon cancer patient group (see methodology section). The number of men was significantly higher in the patients with cancer (45% in the control group vs 68.4% in the CRC group, *p* = 0.018). There were no differences between both groups according to anthropometric variables. Study subjects had a mean BMI of about 28 Kg/m^2^ and a mean age of about 66 years. However, as it was expected, there were statistically significant differences regarding metabolic parameters. The main finding was the difference in 25(OH)D and LINE-1% methylation levels between both groups. LINE-1% methylation values in VAT were lower in the cancer group compared with the control group (63.9% [63.7–64.1 CI] vs. 64.4% [64.1–64.7 CI], *p* = 0.02). The same trend was observed with 25(OH) D values **(**Table 1).

**Table 1:** Anthropometric and biochemical variables of patients with or without CRC

	Control	CRC	*P* value
	*N* = 35	*N* = 55	
BMI (kg/m^2^)	29.03 ± 4.05	27.61 ± 4.01	0.272
Sex(male/female)(%)^a^	45/55	68/32	0.02
Age (years)	64.9 ± 8.8	68.06 ± 8.2	0.067
Weight (kg)	73.70 ± 10.4	73.90 ± 12.5	0.427
Glucose (mg/dl)	117,03 ± 30,9	126,26 ± 47,8	0.091
HOMA-IR^b^	3.64 ± 2.4	1.99 ± 1.8	0.004
Chol (mg/dl)^b^	221.50 ± 42.3	169.10 ± 44.0	< 0.001
HDL-C (mg/dl)^b^	52.30 ± 13.1	40.05 ± 15.4	< 0.001
25-OH-VitD (nmol/l)^a^	36.40 ± 14.9	29.90 ± 13.2	0.022
Insuline (μUI/ml)^b^	12.40 ± 7.4	6.00 ± 4.8	< 0.001
CRP(mg/l)^a^	5.70 ± 3.4	9.30 ± 10.8	0.034
LINE methylation in VAT % ^a^	64.40 ± 1.4	63.90 ± 0.8	0.02

Values are expressed as mean ± SD. ^a^ and ^b^ indicate significant differences between the means of both groups of patients (*p* < 0.05 and *p* < 0.01 respectively)

VAT (visceral adipose tissue)

On the other hand, levels of LINE-1% methylation were higher in men although these differences were not statistically different (64.2% [63.9–64.4] vs 63.8% [63.4–64.2], *p* = 0.08). This trend was shown both cancer (64,09%[63.8–64.4 CI] vs 63,6%[63.2–64.04 CI], p = 0,053) and control group (64,4%[63.5–65.3] vs 64,1%[63.4–64.8], p = 0,52), although there weren’t statistically significant. Regarding age, we did not find any relationship with LINE-1% methylation levels (data not shown), whereas a significant association was found between BMI and LINE-1 methylation (All: r = −-0.257, *P* = 0.014; Cancer group: *r* = − 0.292, *p* = 0.03; Control group: *r* = − 0.295, p = 0.08).

### Relationship between LINE-1% methylation levels, obesity and colorectal cancer phenotypes

To test whether these differences were associated with BMI we classified the subjects according to the following categories: 1) control group without obesity; 2) control group with obesity; 3) cancer group without obesity; 4) cancer group with obesity**.**

Subjects from the control group without obesity showed higher LINE-1% methylation in VAT and serum 25(OH) D levels compared with the other groups (*p* = 0.01 and *p* = 0.035, respectively).

**Fig. 1:**
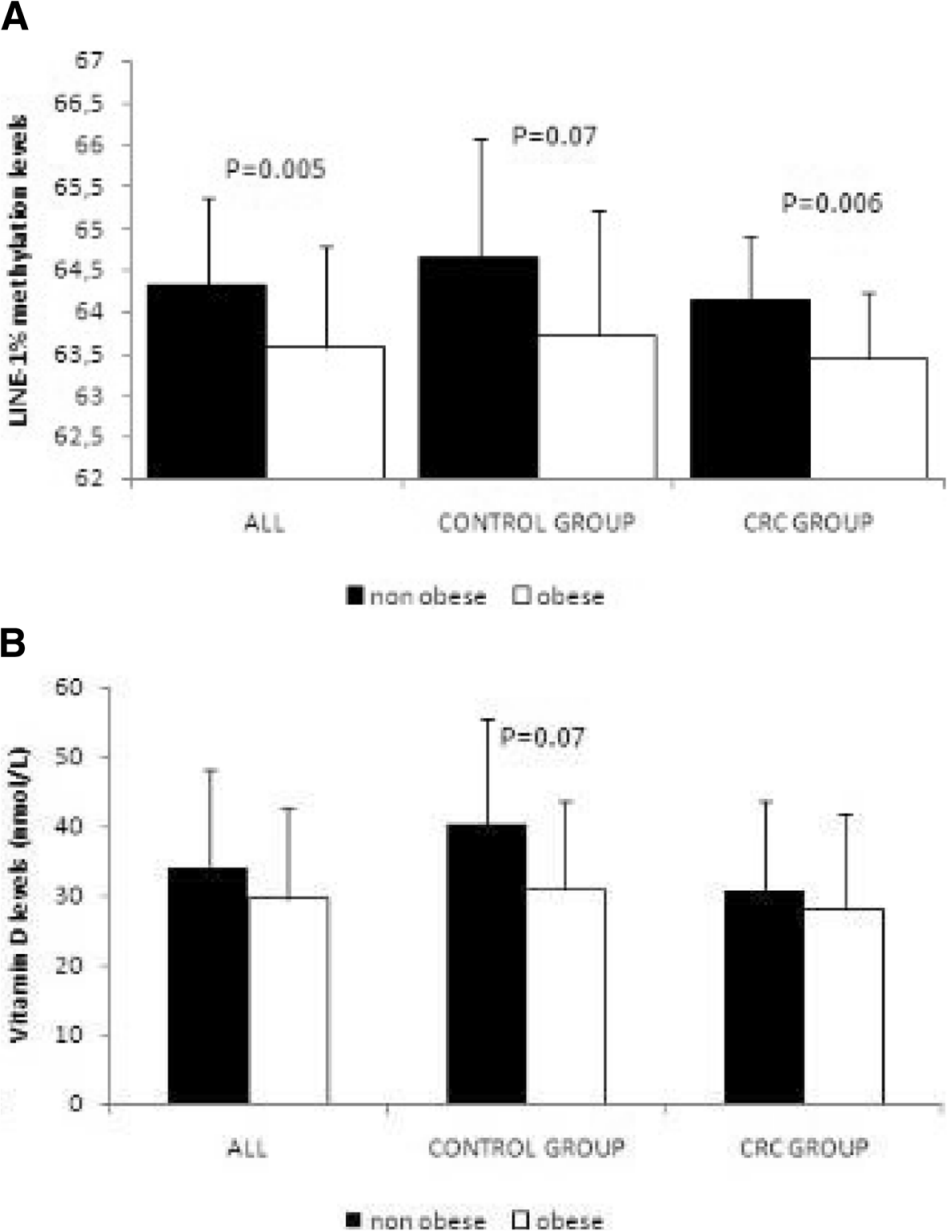
LINE-1% methylation in VAT (**a**) and serum vitamin D (**b**) levels in both groups (control and cancer) according to BMI. Values are expressed as mean ± SD. CRC (colorectal cancer)

In the cancer group, subjects with obesity showed significantly lower levels of LINE-1 methylation than people without obesity, and the same trend was observed in the control group but it was not statistically significant (Fig. 1a). Similar tendency was found in both groups for 25(OH) D values but they did not reach statistical significance (Fig. 1b). In the group of cancer patients, a positive association was observed between LINE-1% methylation levels and vitamin D levels (*r* = 0.463, *p* < 0.001); whereas, a negative association between LINE-1 methylation levels and BMI (*r* = − 0.334, *p* = 0.01) and HOMA insulin resistance index (*r* = − 0.348, *p* = 0.01) were found. However, when we analyzed according to BMI, we observed a positive correlation between serum vitamin D and LINE-1 methylation levels only in the group of subjects with BMI > 30 (*r* = 0.780, *p* < 0.001).

### Predictors of LINE-1%methylation levels in VAT from subjects with and without CRC

Due to the finding about LINE-1 methylation and Vitamin D levels in visceral adipose tissue, a multiple linear regression model was used to test the contribution of the potential variables in the LINE-1% methylation variation. The variance of LINE-1% levels in the cancer group was explained mainly by 25(OH)D (β = 0.460, *p* < 0.001) (Table 2). The model included potential confounding variables such as BMI and HOMA-IR, but these factors were not significant. However, in the control group neither of these variables contributed significantly to the variance of LINE-1 methylation levels **(**Table 2).

**Table 2:** Multivariate linear regression model of predictors of LINE-1 methylation levels in VAT from subjects with and without CRC

	CRC		Control	
	R^2^ = 0.465		R^2^ = 0.235	
	*Standardized β*	*P value*	*Standardized β*	*P value*
Sex (male/female)	−0.222	0.06	−0.088	0.65
Age (years)	0.143	0.21	−0.018	0.92
BMI (Kg/m^2^)	−0.240	0.05	−0.128	0.51
HOMA_IR	−0.172	0.17	−0.397	0.049
Vitamin D (nmol/L)	0.460	0.0003	0.094	0.64

### Predictive value for LINE-1% DNA methylation in VAT as risk marker of CRC

Finally to test the predictive capacity of LINE-1 methylation levels as risk marker of developing CRC, a logistic regression analysis was performed. Regression logistic was done including those known variables related to CRC such as BMI, sex, age and vitamin D. Subjects with higher LINE-1 methylation values had lower risk of developing CRC (OR = 0.53; IC95% =0.28–0.99) in a model adjusted by age, sex, BMI, HOMA-IR and vitamin D levels. However, neither vitamin D nor BMI, contributed significantly to the risk of developing CRC in the present model (Table 3).

**Table 3:** Association of LINE-1 methylation levels with colorectal cancer (CRC) using logistic regression analysis

	0R	IC (95&)	P value
Sex (male/female)	3.8	1.1–13.1	0.034
Age (years)	1.1	1.015–1.204	0.021
BMI (kg/m^2^)	0.94	0.81–1.09	0.423
HOMA-IR	0.65	0.49–0.87	0.004
Vitamin D (nmol/L)	0.97	0.93–1.02	0.284
LINE-1(%)	0.53	0.28–0.99	0.043

Dependent variable: control subjects (0); colorectal cancer subjects (1)

## Discussion

We studied for the first time, the LINE-1 methylation levels in VAT from colorectal cancer. The most relevant finding was the decreased levels of LINE-1 methylation in VAT from cancer patients in relation with the control group, and the strong relationship between LINE-1 methylation in VAT and Vitamin D in the CRC group, being the vitamin D the main variable influencing on global DNA methylation in our study**.**

In this study we wanted to highlight the potential role of adipose tissue in colorectal cancer. Recent results from our group have also shown that adipose tissue could be an important factor in CRC development, and epigenetic mechanisms might be involved. In fact, we have shown that adipose tissue *DNMT3A* mRNA correlates negatively with 25(OH)D and positively with adipose tissue *VDR* and *NfκB1* methylation, suggesting that Vitamin D could be involved in epigenetic modifications in specific genes in adipose tissue by mechanisms involving the DNA-methyltransferase [27].

On the other hand, we observed that the healthy phenotype in this study (control group without obesity) had the highest levels of LINE-1 methylation, supporting the published results in the literature that associate a global hypomethylation with higher instability genomic and in consequence with the susceptibility to develop several non-communicable diseases [30, 31] . Although the absolute values of global DNA methylation are very similar between obese and non-obsese subjects in our study, this difference is very similar to reported in previous studies [32]. There are no many studies measuring LINE-1 DNA methylation in visceral adipose tissue, most of them have been performed in PBMC samples. We published LINE-1 DNA methylation levels in a population cohort of obese and non-obese people. This measurement was done in peripheral blood samples, but the values were very similar to those published in the current work [19]. Usually, the methylation changes in obesity are smaller than those obtained in cancer, but these small differences have biological significance.

Numerous studies have shown an association between a marker for global methylation LINE-1 [33] and worse prognosis and survival in CRC patients [34, 35]. Recently, it has been showed lower LINE-1 methylation levels in patients with CRC [31], but in blood samples other authors have been reported that global DNA hypomethylation was associated with increased risk of cancer [36].

Currently, it is well recognized a global hypomethylation of genomic repetitive sequences in malignant cells, but less is known about the epigenetic pattern in surrounding tissues. In fact, we have also observed a hypomethylation of LINE-1 in tumor tissue compared to adipose tissue in the CRC group (data not shown). This is also demonstrated for some authors in non tumor tissues, where they showed decreased in the global DNA methylation related with significantly associated with worse prognostic in patients with CRC cancer [37].Similar results have also been found by *Suter* et al [14], suggesting that hypomethylation could begin a process in which some genes are dysregulated in the first steps in the developing of CRC.

To our knowledge, this is the first study evaluating the global epigenetic pattern in VAT from CRC patients. Due to the close relationship between obesity and cancer, we hypothesized that epigenetic regulation in the adjacent VAT, could be involved in the colorectal carcinogenesis.

Evidence exists that dietary components may influence the inflammatory process and the risk of developing CRC [38]. Evidence exists that dietary components may influence the inflammatory process and the risk of developing CRC [38]. On the other hand, several bioactive food components, have been shown to influence in the hypomethylation of LINE-1, which has been associated with an increased risk of several cancers [18]. However, conflicting findings have also been observed depending on whether the study of the global DNA hypomethylation was made in biopsies from different areas of colon or in the PBMCs [39].

In the last years, vitamin D is acquiring great importance in the link obesity and cancer [40]. vitamin D levels have been inversely associated with cancer risk in several studies [25]. Likewise, an association between obesity and low vitamin D status has been shown [40]. We observed lower levels of Vitamin D in the cancer patients compared to the control group. Furthermore, a strong positive correlation between LINE-1 methylation and Vitamin D levels were found in the CRC group with obesity. This finding highlights the potential role of Vitamin D as an epigenetic factor regulating the global DNA methylation pattern especially in subjects with obesity.

Vitamin D system has a very important role to maintain the genome stability [41], through different mechanisms protecting against oxidative stress and aging processes [42]. There are not many studies evaluating the association of this important nutrient on global DNA methylation status. Tapp et al. [43], showed that LINE-1 methylation of rectal mucosa from healthy subjects was influenced by vitamin D among other factors. We observed the same association but only in the CRC group. Other authors have shown conflicting results. For example, *Hübner* and colleagues have not showed any effect of the vitamin D supplementation seems to have no effect on LINE-1 methylationin the global methylation in patients with moderate VD deficiency [44]. However, this intervention studies with VD has been made only in blood cells, so it does not mean that have any effect in other tissue methylation status of specific genes. Our results showed that in CRC subjects, VD was the only variable studied that correlated positive significantly with LINE-1 in the cancer group but not in the control group. These results would be consistent with the study by *Pufulete* et al [45], where found a different effect of vitamin supplementation in patients with colorectal adenoma. We only observed this effect in the cancer group, suggesting that patients with cancer might respond to vitamin supplementation in a different way, but associated with aberrant changes in LINE-1 DNA methylation.

## Conclusions

In conclusion, we found that CRC patients had lower levels of LINE-1 methylation than control group in VAT samples. This LINE-1 methylation pattern was positively associated with serum Vitamin D levels, pointing to a significant association of this nutrient with DNA methylation profile. Finally, we could suggest that the VAT of CRC patients is suffering epigenetic changes that could contribute to the development or appearance of the tumor, and vitamin D could be involved in this epigenetic regulation.

## Abbreviations

25(OH)D: Serum 25-hydroxyvitamin D
BMI: Body mass index
CCR: Colorectal cancer
CRP: C-reactive protein
HDL: High density lipoprotein
HOMA-IR: Homeostatic model assessment insulin resistance (IR)
LINE-1: Long Interspersed Nucleotide Element 1 (LINE-1)
SAT: Subcutaneous adipose tissue
VAT: Visceral adipose tissue
VDR: Vitamin D receptor
